# Systemic and prophylactic intrathecal chemotherapy for primary adrenal lymphoma: A retrospective study of 20 case reports

**DOI:** 10.1097/MD.0000000000015662

**Published:** 2019-06-14

**Authors:** Lei Yuan, Lu Sun, Jian Bo, Quanshun Wang, Yu Zhao

**Affiliations:** a Department of Hematology; b Department of Pathology, Chinese PLA General Hospital, Beijing, China.

**Keywords:** adrenal insufficiency, primary adrenal lymphoma, prognosis, retrospective analysis

## Abstract

Primary adrenal lymphoma (PAL) is a rare entity of lymphoma with dismal prognosis using systemic chemotherapy. More clinical reports are needed to guide the treatment for PAL.

We performed a retrospective analysis of 20 patients diagnosed with PAL who presented to our center between January 2005 and January 2014.

Median age at presentation was 48 years (range: 27–73) with a male-to-female ratio of 7:3. Bilateral and right-sided adrenal involvement were seen in 11 of 20 and 7 of 20 patients, respectively. Adrenal insufficiency (AI) was seen in 6 of 10 evaluated patients. Diffuse large B cell lymphoma (DLBCL) was the most common immunophenotype (85.0%). Two patients died due to rapid disease progression before treatment. Two patients received autologous stem cell transplantation as consolidation therapy. All patients received prophylactic intrathecal chemotherapy. The estimated 5-year overall survival (OS) and progression-free survival (PFS) were 52.5% [95% confidence interval (95% CI: 28.2–72.0)] and 53.2% (95% CI: 29.0–72.5), respectively.

These findings suggest that PAL should always be considered in differential diagnosis of adrenal mass with AI. Despite the contrasting previous reports, long-term prognosis of PAL is not necessarily inferior to that of non-Hodgkin lymphoma in general.

## 1 Introduction

Adrenal gland involvement has been radiologically reported in up to 5% of all cases of systemic lymphoma; with autopsy confirmation, this number increases to 25% for non-Hodgkin lymphomas (NHLs).^[1–4]^ However, primary adrenal lymphoma (PAL) is rare, with slightly more than 250 cases currently described in the English-language literature.^[1,4]^ In current classifications, there is not yet a consensual definition of PAL, with most reports and experts defining PAL as a histologically proven lymphoma involving at least 1 adrenal gland with 2 additional criteria: there is no antecedent history of lymphoma elsewhere, and lymph node or other organ involvement is detectable, but adrenal lesions are unequivocally dominant.^[4]^ A systematic review concluded that PAL predominantly affects elderly men.^[1,4,5]^ In addition, bilateral adrenal involvement has been described in about 70% of cases. The most common histological subtype (70%) of PAL is nongerminal center type diffuse large B cell lymphoma (DLBCL).^[1–7]^

No standard therapeutic protocol for PAL has yet been established, and several reports have used combination therapy, including surgery and chemotherapy.^[1,2,4,8–11]^ For a long time, the prognosis of PAL was dismal given high incidence of disease recurrence and early death related to delayed diagnosis. Ichikawa et al^[12]^ argued that perhaps one of the reasons for the poor prognosis is that many patients previously treated with chemotherapy did not receive rituximab and central nervous system (CNS) prophylaxis, which may have decreased overall survival. In addition, only with accumulated recognition on this special subentity of aggressive lymphoma can early and accurate diagnosis become possible.

However, the majority of published articles about PAL are case reports or case series study with only a limited number of patients. Here, we report the clinical features, pathological and imaging features, and improved treatment outcome of 20 patients with PAL who presented at our center did receive regular systemic chemotherapy combined with CNS prophylaxis and additional rituximab for PAL of DLBCL.

## 2 Patients and methods

### 2.1 Patient population

Between January 2005 and January 2014, 20 patients with PAL were diagnosed at Chinese PLA general hospital in Beijing, China. We retrospectively reviewed the medical records of these patients for the current study after approval from the institutional review board. Defining PAL as a histologically proven lymphoma involving at least 1 adrenal gland with 2 additional criteria: there is no antecedent history of lymphoma elsewhere, and lymph node or other organ involvement is detectable, but adrenal lesions are unequivocally dominant. A diagnosis of adrenal insufficiency (AI) was based on the cortisol value <5 μg/dL at 8 am (following oral dexamethasone 1 mg taken late the previous evening) and the presence of 1 of the 2 PAL-specific criteria mentioned in the Introduction section.

### 2.2 Treatment protocol

**Figure 1:**
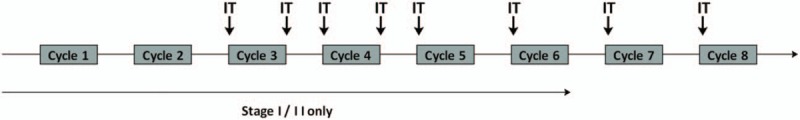
The therapeutic scheme of systemic and intrathecal chemotherapy. IT = intrathecal chemotherapy).

As illustrated in Fig. 1, all patients with DLBCL received prednisone (90 mg orally days 1–5), vincristine (1.4 mg/sqm i.v. day 1), cyclophosphamide (750 mg/sqm i.v. day 1), or doxorubicin (45 mg/sqm i.v. day 1) along with rituximab (375 mg/sqm i.v. day 0) (R-CHOP regimen). Patients with T-cell origin were treated with prednisone (90 mg orally days 1–5), vincristine (1.4 mg/sqm i.v. day 1), cyclophosphamide (750 mg/sqm i.v. day 1), doxorubicin (45 mg/sqm i.v. day 1), and L-asparaginase (5000 IU s.c. day 1) (CHOP-L). Patients with Hodgkin lymphoma (HL) received a regimen of doxorubicin (25 mg/sqm i.v. days 1 and 15), bleomycin (10 units/sqm i.v. days 1 and 15), vinblastine (6 mg/sqm i.v. days 1 and 15), and dacarbazine (375 mg/sqm i.v. days 1 and 15) (ABVD). Patient with PAL of unknown origin received the CHOP regimen only. CHOP regimen was given at 3-week interval, whereas the ABVD regimen was given at 4-week interval. Response to treatment was evaluated every 2 cycles of chemotherapy. All patients received prophylactic intrathecal chemotherapy with methotrexate (MTX) of 10 mg and cytarabine (Ara-C) of 50 mg combined with dexamethasone (Dex) of 5 mg, twice around cycle 3 to 4, and once in cycle 5 to 8.

### 2.3 Evaluation of efficacy and toxicity

Rate of complete response (CR), partial response (PR), stable disease (SD), probability of progression-free survival (PFS), and overall survival (OS) was analyzed.^[13]^ Response was determined after 2 cycles of chemotherapy by contrast-enhanced computed tomography (CT) or whole body positron emission tomography-computed tomography (PET-CT) scan. During the 5-year follow-up period, all patients underwent clinical and radiographic evaluations every 3 to 6 months.

### 2.4 Statistical analysis

OS and PFS were analyzed with Kaplan–Meier method. OS was calculated as the time of diagnosis until the last follow-up or until death. PFS was calculated as the time from diagnosis until the last follow-up point for event (relapse) for alive patients or death.

## 3 Results

### 3.1 Patients characteristics

Baseline characteristics, pathological and imaging features, and treatment outcomes of 20 patients with PAL diagnosis are described in Table 1. Briefly, median age at presentation was 48 years (range: 27–73 years) with a male-to-female ratio of 7:3. All patients with PAL presented with localized abdominal pain. B-symptoms (weight loss, low-grade fever) were observed in 12 of 20 patients. Six of 10 evaluated patients had AI, depending on the extent of adrenal involvement.

**Table 1:**
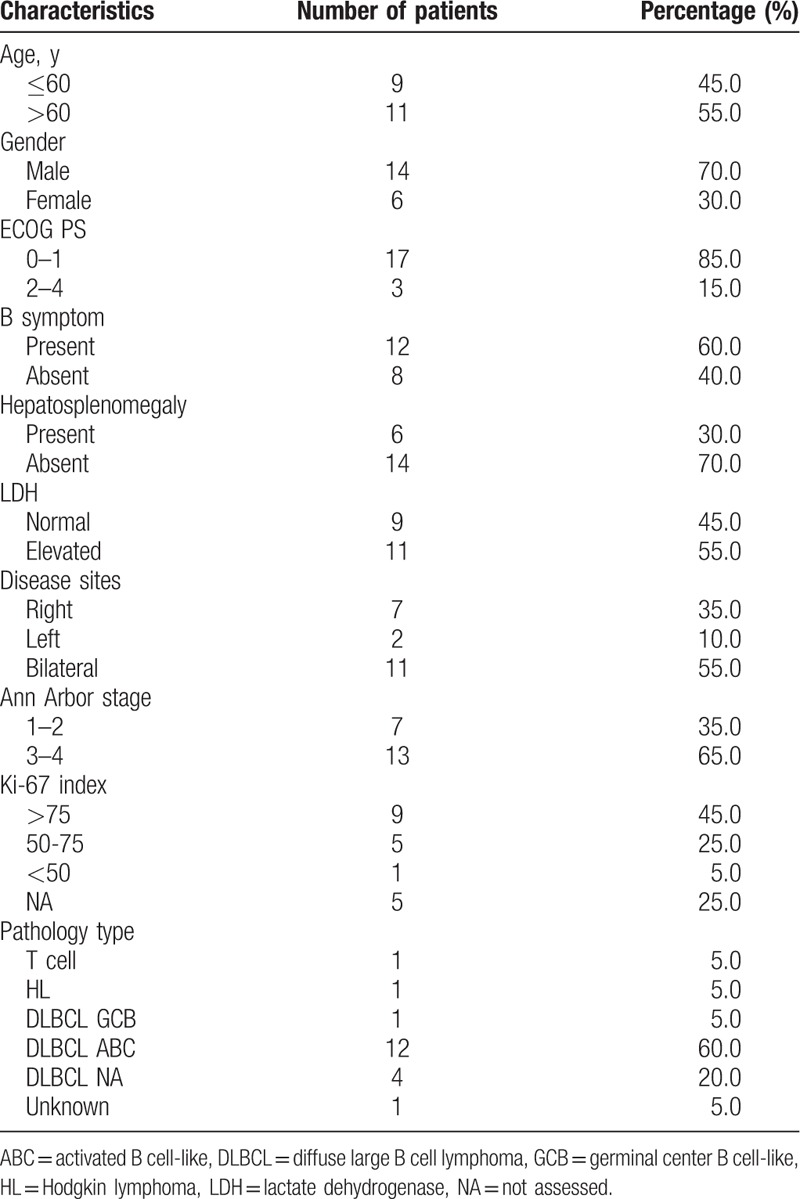
Clinical, pathological, and imaging characteristics of 20 patients with primary adrenal lymphoma.

### 3.2 Imaging features

^18^F-fluorodeoxyglucose positron emission tomography-computed tomography (^18^F-FDG PET-CT) and magnetic resonance imaging (MRI) were available in 11 and 5 patients, respectively. Two patients underwent both evaluations. Bilateral adrenal gland involvement was observed in 11 of 20 patients (55.0%). In patients with unilateral disease, right and left involvement was seen in 7 and 2 patients, respectively. ^18^F-FDG PET-CT showed intense metabolic activity in adrenal masses as well as involvement of some regional lymph nodes. Follow-up ^18^F-FDG PET-CT was used to assess the metabolic as well as morphological response to treatment.

### 3.3 Pathological diagnosis

Final diagnosis of PAL was confirmed by whole body imaging, histopathological examination, and immunohistochemistry of adrenal tissue obtained through biopsy. DLBCL was observed in 17 patients; the remaining patients included 1 with PAL of T-cell origin, 1 of HL, and 1 of unknown origin. None of the patients with PAL had any prior history of adrenal autoimmune diseases.

### 3.4 Treatment outcome

All patients were treated with systemic chemotherapy as well as regular intrathecal chemotherapy. Two patients died before starting chemotherapy due to rapid disease progression. Two patients with PR on interim evaluation received autologous hematopoietic stem cell transplantation (Auto-HSCT) as consolidation therapy. Two patients with stage 2 at diagnosis received focal irradiation therapy after 4 courses of chemotherapy.

**Figure 2:**
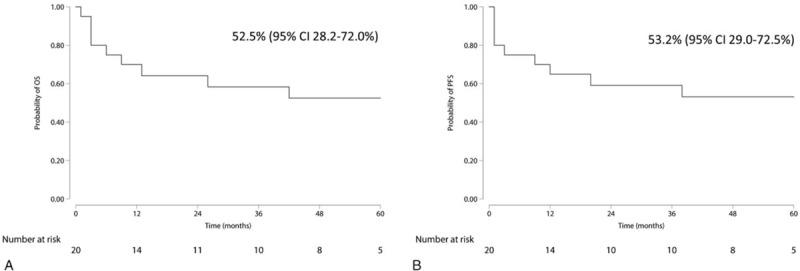
Overall survival (A) and progression-free survival (B) of 20 primary adrenal lymphoma (PAL) patients.

**Figure 3:**
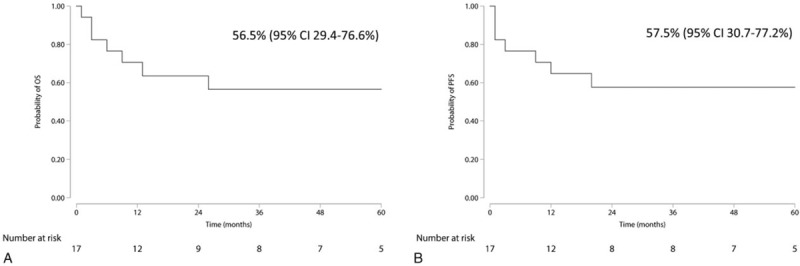
Overall survival (A) and progression-free survival (B) of 17 primary adrenal lymphoma (PAL) patients of diffuse large B cell lymphoma.

After the first-line treatment regimen, 9 patients achieved CR, 6 with PR, and 2 presented with SD. Two patients died of infection related to chemotherapy. Six patients died of primary disease. One patient was lost after a period of 12-month follow-up under PR. The estimated 5-year OS and PFS of the whole cohort were 52.5% [95% confidence interval (95% CI): 28.2–72.0] and 53.2% (95% CI: 29.0–72.5), respectively (Fig. 2). For 17 PAL of DLBCL, the estimated 5-year OS and PFS were 56.5% (95% CI: 29.4–76.6) and 57.5% (95% CI: 30.7–77.2), respectively (Fig. 3).

## 4 Discussion

In this study, we report the clinical presentation and treatment outcome of 20 patients with PAL, a rare subtype of lymphoma, who presented to our center during the last 10 years. To the best of our knowledge, this is the largest single-center case series of PAL in the English-language literature. Similar to previous reports, men were predominantly affected with a male-to-female ratio of 7:3, which was slightly lower than the 7:1 value previously reported.^[4]^ In addition, our patients were comparatively younger (median age at presentation of 48 years) than those enrolled in previous related studies.^[4]^ In patients with unilateral adrenal involvement, localized abdominal pain with or without B symptoms was the most common initial presentation not accompanied with other symptoms. Unlike previous studies, AI was the dominant symptom in patients with bilateral disease of PAL in our study.^[1–4]^ PAL occurs in association with autoimmune diseases in 20% of cases. However, 7 cases (35%) had a history of thyroid autoimmune disease, but no cases of such disease in adrenal gland were observed. The latter one was possibly due to the nature of the retrospective analysis.

AI is not a sensitive factor to rule out the diagnosis of PAL. Given abundant adrenal reserve, destruction in at least 90% of the adrenal parenchyma was required to cause AI. A relatively large proportion of PAL patients had normal adrenal function at diagnosis. And in the present study, similar to that reported previously, AI was only confirmed in 6 of the 10 evaluated patients.^[10,14–16]^ Besides, PAL cells also secrete cytokines and produce paracrine effects that may create a microenvironment conducive to the development of AI.^[4]^ Thus, this may at least partly explain the reason for the presence of AI in PAL patients with smaller adrenal masses. Hence, in agreement with previous conclusion, combining with other nonspecific features and imaging characteristics, it would be rather challenging to make early diagnosis of PAL.

It has been postulated that adrenal autoimmune disease and direct infiltration of neoplastic lymphoid cells are the leading causes of adrenal hypofunction.^[8]^ However, AI in other reports has been identified to be more common in bilateral PAL than in unilateral PAL or adrenal metastasis.^[7,14,17,18]^ In the present study, PET-CT scan of the patients showed positive evidence of occupational masses, which strongly implied that AI in such circumstances might be the direct result of neoplastic infiltration. Thus, although imaging examinations were not sufficiently powerful in diagnosing PAL, it is still predictive and necessary.

Similar to current knowledge, DLBCL was the most common subtype of PAL in our series (17/20, 85%).^[4]^ Of the 13 evaluated DLBCL patients, only 1 originated from the germinal center, which may in part explain the poor prognosis of PAL. Almost all previous reports have illustrated that durable remission after systemic chemotherapy is rare with higher incidence of disease recurrence in either primary site or CNS, despite a good initial response.^[1,4,5,8,10,12,16,19–23]^ A recent review showed poor prognosis for PAL with 3-, 6-, and 12-month survival rates of only 67%, 46%, and 20% of patients, respectively.^[4]^ However, in our series, an OS plateau still existed, indicating that patients achieving CR or stable PR would have a better outcome. Another study reported 2-year OS and PFS rates of 68% and 51%, respectively, when treated with the R-CHOP regimen.^[1]^ Outcomes in this study were better than those reported in earlier studies, suggesting the benefit of adding rituximab to the therapy regimen combined with regular and sufficient prophylactic intrathecal chemotherapy; this likely also contributed to the superior outcome in the present study.

Factors contributing to these results also include the short duration of onset to diagnosis, intensive chemotherapy, low lymphoma burden, and sensitivity to chemotherapy as previous report.^[4]^ In our study, we reviewed carefully in the case record system and found that all patients had an initial symptom of back pain and obtained their diagnosis of PAL within 6 months (data not shown). Meanwhile, 2 patients whose disease were not sensitive to the first-line chemotherapy received upfront auto-HSCT and maintained CR until this report. We also have to acknowledge that this superior outcome may be due to a relatively younger population and lower lymphoma burden in our cohort. A systematic review of PAL concluded that elevated serum LDH levels and involvement of multiple extranodal sites were associated with worse outcome. And administration of chemotherapy was a significant predictor of longer survival.^[4]^ At present, given no published prospective study on PAL, comparison on these factors between our study and others is impossible. Thus, further larger population-based clinical prospective studies are needed to investigate these aspects.

However, the present retrospective analysis-based study has several limitations. A small number of cases and an incomplete patients’ characteristics given of information from our case record system made it impossible to identify reliable prognostic factors. Moreover, the relatively heterogeneous therapeutic protocol and a lack of control would negatively influence the confidence of the final outcome.

## 5 Conclusion

Taken together, in clinical practice, patients with adrenal mass, particularly those with bilateral involvement or AI, may present with PAL. PAL is a rare aggressive subentity of lymphoma with a historically poor prognosis. However, early diagnosis, immediate treatment, and intensive chemotherapy, including rituximab regimen combined with regular and sufficient prophylactic intrathecal chemotherapy, would yield an improved outcome.

## Acknowledgment

The authors thank all the physicians and nurses at the Department of Hematology of Chinese PLA General Hospital for their contribution.

## Author contributions

Protocol design: Q.S.W., Y.Z.; Data collection: L.Y., L.S., Y.Z.; Data analysis: L.Y., J.B.; Manuscript drafting: L.Y., Y.Z.

**Conceptualization:** Quan-shun Wang.

**Data curation:** Lu Sun, Jian Bo.

**Supervision:** Yu Zhao.

**Writing – original draft:** Lei Yuan.

**Writing – review & editing:** Yu Zhao.
